# Cleaner wrasse failed in early testing stages of both visual and spatial working memory paradigms

**DOI:** 10.1007/s10071-025-01959-w

**Published:** 2025-06-04

**Authors:** Leonore Bonin, Héctor M. Manrique, Redouan Bshary

**Affiliations:** 1 Biology Institute, Université de Neuchâtel, Neuchâtel, Switzerland; 2 Department of Psychology and Sociology, University of Zaragoza, Zaragoza, Spain

**Keywords:** Working memory, Animal cognition, Cleaner wrasse, Executive functions, Behavioral ecology, Ecological approach to cognition

## Abstract

Working memory (WM), an attention-based short-term storage system responsible for the manipulation and integration of past knowledge with present information for goal-directed behavior, is a key executive function and a principal predictor of general intelligence. Despite its importance, WM has not been a major research topic in animal behavior. Here, we first summarize key ideas related to WM from the social sciences for interested colleagues. Given that past methodological inconsistencies have led to mixed results and conclusions across various species, we then designed experiments that incorporate the critical components of WM, facilitating cross-species comparisons and accounting for potential ecological influences. We present such experiments on WM in an ectothermic vertebrate, the cleaner wrasse (*Labroides dimidiatus*), which faces environmental challenges potentially requiring complex cognitive adaptations. Overcoming several experimental challenges, we consistently obtained negative results across multiple experimental paradigms. As our experiments were specifically designed to test WM, our negative results call into question previous studies in other fish species that provide evidence for WM using different paradigms. More specific tests for WM should be developed to confirm the presence or absence of this executive function in other ectotherm vertebrates. The absence of WM may be a key factor underlying the significant encephalization gap between ectotherm and endotherm vertebrate species.

**Supplementary Information:**

The online version contains supplementary material available at 10.1007/s10071-025-01959-w.

## Introduction

Among vertebrates, brain size relative to body size exhibits considerable variation (Jerison 1969). On average, endotherms possess brains that are approximately ten times larger than those of ectotherms when corrected for body size (Jerison 1969). Given the high energetic demands of brain tissue (Fonseca-Azevedo and Herculano-Houzel 2012; Sukhum et al. 2016), an important question arises: what advantages do larger brains confer to endotherms? One hypothesis is that endotherms possess a more extensive cognitive toolkit compared to ectotherms. While both groups exhibit basic learning strategies that allow them to form new stimulus-stimulus (Pavlovian conditioning, Pavlov 2010) and stimulus-response-consequence associations (operant conditioning, Skinner 1937), and/or learn new adaptive responses following the presentation of a reward (Thorndike 1927), these strategies do not require flexible manipulation of mental objects. Whether ectotherms are capable of "complex cognition", e.g., cognitive abilities involving the executive functions of the brain (EFs, also called executive or cognitive control) (Knauff and Wolf 2010) remains poorly understood and warrants further exploration.

A complementary hypothesis regarding the differences in brain size between endotherms and ectotherms relates to the sophistication of these EFs. These higher-level cognitive skills are central to complex cognition, as they involve building intricate interactions among mental processes of varying complexity (Diamond 2013). The three primary EFs—inhibition, cognitive flexibility, and working memory—“are recruited when it would be ill-advised, insufficient, or impossible to go on autopilot or rely on instinct or intuition, such as when presented with novel, unanticipated challenges” (Diamond 2020, p. 225). Although research on EFs in ectotherms is scarce, some studies suggest that fishes perform well in tasks assessing cognitive flexibility as shown in reversal learning (Aellen et al. 2022; Parker et al. 2012)—where performance correlates positively with brain size in primates (Deaner et al. 2007)—and demonstrate proficiency in inhibitory control tasks, such as delayed gratification paradigms (Aellen et al. 2021) and detour tasks (Lucon-Xiccato and Bisazza 2017; MacLean et al. 2014).

In this study, we investigated the role that working memory (WM), a critical EF, could play in ectotherms’ cognition. There is a vast literature on WM in the social sciences. Research on WM includes comparative developmental psychology, i.e. comparisons between children and great apes. While definitions of WM have become more detailed and robust in recent years, there is no clear consensus on how to test for WM (Manrique et al. 2024). As we consider such aspects critical for solid and repeatable research on WM in animals, we first provide a brief summary of definitions and methodological considerations, inspired by Read et al. (2022) and Manrique et al. (2024), to help readers follow the logic of our work.

## WM definition and importance

WM is among the most well-studied EFs in humans, with extensive research dating back more than 80 years to Miller et al. (1960), who referred to “the memory we use for the execution of plans as a kind of quick access working memory” (p. 65). While numerous models of WM have been proposed, we focus on the Embedded-Processes model of Cowan (1988, 1995), which has undergone recent refinements (Cowan et al. 2024), and it is not reliant on language. We redirect readers to appropriate literature for descriptions and debates about other models (e.g. Baddeley and Hitch 1974; Chai et al. 2018; Conway et al. 2003; Cowan 1988, 1995; Cowan et al. 2024; Fuster and Bressler 2012; Logie et al. 2021; Morrison 2005).

The Embedded-Processes model consists of a focus of attention (FoA) within a temporarily activated portion of the long-term memory (aLTM). Cowan et al. (2024) defined the FoA as a “coherent representation of several separate items or ideas guiding current thoughts and actions” (p. 186) and the aLTM as “the part of memory from which information is in a heightened state of accessibility” (p.186). Hence, in this model, a new stimulus can enter either consciously with the intervention of the central executive, or unconsciously into the aLTM. Part of the information related to this stimulus directly enters into the FoA and reaches awareness. Thanks to the central executive, some knowledge stored in the LTM can be brought into the FoA to “guide focus”. By combining them, new “chunks” are created and can move from the FoA to the aLTM, and finally to the LTM where this new acquired knowledge will remain stored. In line with this model, Manrique et al. (2024) defined WM as a “brain system that provides us with temporary short-term storage and management of perceptual or other information (...), which we need for efficiently (...) carrying out, and updating, such complex cognitive tasks as mental reading, reasoning, forecasting, manipulation, (...)” (p. 1). This is the definition we followed here. In other words, WM is a short-term storage system responsible for the manipulation and integration of past knowledge with present information for goal-directed behavior. According to Cowan’s (1988, 1995) Embedded-Processes model, WM is an attention-based mechanism. The manipulative aspect of WM distinguishes it from short-term memory.

WM is of potential importance because it has been linked to many cognitive abilities. For example, training WM can improve crystallized intelligence (Alloway and Alloway 2009). WM is also a major predictor of the general intelligence factor ‘*g’,* i.e. the positive correlations among individual performances across different cognitive tests (Burkart et al. 2017), in mice (Kolata et al. 2005)—an endotherm species displaying an average brain size-body size relationship (Tartarelli and Bisconti 2006)—and in humans (discussed in Conway et al. 2003). WM is also correlated with numerous other cognitive and perceptual abilities in humans (Ackerman et al. 2002; Engle 2010; Unsworth et al. 2015), and the prospective aspect of WM is key to efficient tool crafting (Manrique and Walker 2017). At present, the only experiment that has tested the general intelligence factor *g* in fish yielded negative results (Aellen et al. 2022), suggesting that their WM might indeed be more primitive and/or not used in a domain-general way. Alternatively, ectotherms might have WM capacities similar to endotherms, but these capacities could be modular and only elicited under ecologically relevant circumstances.

## How to test for WM?

There are not only multiple definitions of WM, but also different opinions on how to test it. Cowan et al. (2024) argue that “Discrepancies between methods or definitions often underlie discrepancies between results rather than unreliability of evidence” (p. 186), highlighting the importance of developing a clear and common methodology. Testing WM in animals presents distinct challenges, especially in the absence of language. Tasks commonly used in human studies, such as verbal WM tasks (Hu et al. 2019), are not feasible in animal studies. As a reminder, the main characteristics to consider when designing a WM experiment are: manipulation of information (combination of past knowledge and present information), time delay (i.e. short-time storage), capacity-limits (i.e. very limited storage capacity, unlike for instance sensory memory), goal-directedness (i.e. a task must be accomplished), and the sensitivity to disturbance as it is based on attention. Key criteria that help to differentiate WM from other types of memory, which should therefore be carefully considered during the conceptualization of WM tasks, are presented in Table 1.

**Table 1:** Characteristics of various types of memory to highlight the specifics of WM and help differentiating between them

Type of memory		Definition	Time interval	Source
Short-lived storage	Sensory memory: Iconic (visual), echoic (auditory), haptic (touch)*	Time-limited storage of large amount of raw perceptual information, non-manipulative	Milliseconds to 4 s (echoic memory)	Atkinson and Shiffrin (1968), Darwin et al. (1972), Irwin and Thomas (2008), Miller et al. (1960), Phillips (1974), Sligte et al. (2010), Sperling (1960) and Wang et al. (2022)
	Working memory	Attention-based short-term storage used for problem-solving in the present thanks to the manipulation and combination of temporarily activated information from LTM with present information	Few seconds to several minutes**	e.g Baddeley, (1986), Cowan (1995), Logie et al. (2021) and Sligte et al. (2010)
	Short-term memory	Similar to working memory in that the incoming sensory information is held active; however, the information is neither transformed nor manipulated***	Few seconds to several minutes**	e.g. Aben et al. (2012), Baddeley and Hitch (1974), Kail and Hall (2001) and Sligte et al. (2010)
Long-term memory (LTM)	Explicit (declarative****): episodic memory, including prospective memory	The ability to talk about episodes (i.e. specific events) of life, to “mentally time travel”. It requires a sense of self, subjective time, and autonoetic awareness (conscious awareness that something happened to us at a specific time in the past). When the self is projected in the future to anticipate future events, we talk about prospective memory (i.e. future thinking)	Minimum of 15 s after acquisition of information (Atkinson and Shiffrin 1968)	Manrique and Walker (2017) and Tulving (1972, 2002)
	Episodic-like memory	Due to the absence of language, it is impossible to ascertain whether autonoetic awareness accompanies recollection. Therefore, in nonhuman animals, what can be reliably ascertained is whether they recall the *what*, *where* and *when* of a past experience; hence it is also called the what/where/when memory (WWW-memory).		Pause et al. (2013), Suddendorf and Busby (2003) and Tulving (1972, 2002)
	Explicit (declarative****): Semantic memory	Remembering general facts learned in the past (e.g. the country names on a map), it is the storage of factual information		Manrique and Walker (2017)
	Implicit (non-declarative ****)	Memory system used in everyday skills performed automatically without conscious recall		Manrique and Walker (2017), Squire (2004) and Squire and Dede (2015)

*Those are the main types of sensory memory, more are referred to inWan et al. (2020)

**Not clearly defined, highly dependent on the experimental paradigm and/or the discipline (e.g., Neuroscience *vis a vis* Psychology)

***Although the separation between STM and WM is still subject to debate among the most knowledgeable authors (e.g. Baddeley and Hitch 1974; Cowan 2008, 2019; Hitch et al. 2025), we consider it important to highlight the manipulative aspect, hence our separation.

****Declarative or non-declarative refers to the ability to express is, using spoken or unspoken (i.e. signing or writing) languages. Hence, this terminology does not apply to animal studies.

Additional information and details can be found in Squire (2004), Squire and Dede (2015)

There is still much ongoing debate about the different types of memory that we can identify as a function of the time elapsed, the contents stored, or the brain structures and circuits involved. While our proposed classification remains open to discussion, we believe it provides a useful framework for analyzing and comparing results across different nonhuman species and for clearly conceptualizing working memory

Keeping in mind the above-listed criteria, results from various non-verbal tasks aimed at testing for WM must be interpreted with caution if they lack critical elements such as, for example, a manipulative or problem-solving aspect. Such methodological issues are readily apparent in some spatial tasks. For example, in zebrafish, free exploration of a Y-maze and spontaneous alternation has been taken as evidence of WM (Fontana et al. 2021). Similarly, testing for the Piagetian stage 4 of object permanence abilities has also been taken as evidence of WM in cichlids (Guadagno and Triki 2024) and in dogs (with time delay, Fiset et al. 2003) even though recovering the object only requires retrieval of information rather than the mental manipulation of temporarily stored information. This criticism does not apply to higher stages of object permanence (i.e., Piagetian stages 5 and 6) where the position of the object must be subsequently updated, thus imposing higher demands on WM. These latter stages have already been used to test for WM in corvids and psittacids (Pepperberg and Funk 1990; Zucca et al. 2007).

Zebrafish performance on a delayed-match-to-sample (DMTS) task with time intervals of 3 and 4 s has been taken as evidence of WM (under certain circumstances and with the performance dropping at 5 s delay, Bloch et al. 2019). The same paradigm has been applied to honeybees (Zhang et al. 2005) yielding positive results for delays between around 1 and 9 s. However, these types of tasks rely heavily on visual perceptual information and could be seen as “recognition tasks” (Bachevalier and Mishkin 1986; Zola et al. 2000) which could be solved by relying on non-conscious processes, such as familiarity (Yonelinas 2002). It would be crucial to test whether success in such a task depends on recollection or simply familiarity (difference explained in Yonelinas 2002). Whether recognition of a previously presented stimulus is a reliable measure of WM is debatable, but success in DMTS tasks as performed in these two studies does not require subjects to engage with and manipulate information, which is a major criterion to confidently differentiate WM from other short-lived storage (Table 1). The fact that species do not clearly differ from each other when the delays are short in a DMTS task (Lind et al. 2015) further suggests that it is not an appropriate assessment of WM. Recent studies have also identified methodological limitations when testing WM in nonhuman primates, such in the “limited-hold memory task” applied to measure memory in chimpanzees (Inoue and Matsuzawa 2007). This task may instead rely on imagery mechanisms that do not tap into WM (Abdi et al. 2022; Manrique and Walker 2017; Miller and Peacock 1982; Özbaydar 2012; Read et al. 2022). Many other methodological and conceptual limitations have been identified in relation to the measurement of WM in great apes, which are discussed extensively in Read et al. (2022) and Manrique et al. (2024).

Another important consideration when testing WM involves selecting the appropriate time interval separating the acquisition from the retrieval of information (Table 1; Carruthers 2013). This is not trivial, as WM is connected not only to other short-term storages, but also, critically, to the activation of portions of information stored in long-term memory. Whether we agree or not on the use of DMTS as paradigms assessing WM, a previously-cited meta-analysis pointed out the variation in results depending on the time delay in multiple species (Lind et al. 2015), further emphasizing the importance of controlling for these factors. Further involvement of long-term memory in the resolution of a task might also depend on whether participants have time to combine the information (Morey and Cowan 2018). Sensory memory, the non-manipulative short-time storage (Table 1), is known to last from milliseconds to a maximum of 4 s in humans (Darwin et al. 1972; Sperling 1960). Previous studies have often failed to explain the specific time interval chosen to elicit WM sufficiently; measurements can range from 0 s (e.g., Fiset et al. 2003) to 15 min (Fontana et al. 2021), which can easily lead to confusion and mixed results. Additionally, it has been proposed that sensory memory (e.g., iconic) and short-term memory can support performance when the delays are shorter and when the response does not require any precision (for instance, in delayed matching-to-sample experiments as implemented in zebrafish and honeybees in the previously discussed studies Bloch et al. 2019 and Zhang et al. 2005) (Sligte et al. 2010). WM would be recruited if/when the waiting delays between sample and test patterns are longer and/or the response requires the retrieval of more detailed information (Sligte et al. 2010). In spatial tasks, such as in maze experiments (Kraeuter et al. 2019), a potential shortcoming is that the time intervals used, and the number of items to be remembered, may link performance more to long-term WM (Carruthers 2013), in addition to potential alternative strategies that can lead to success without requiring WM (e.g., Hughes and Blight 1999; Still 1966).

A final key feature of WM is the involvement of attention, which is notably highlighted in Cowan's Embedded-Processes model (1988, 1995). Sensitivity to disturbance is a critical characteristic of WM testing (e.g., Angelopoulou and Drigas 2021; Cowan 1988; Cowan et al. 2024) that has been largely overlooked in previous studies (e.g., Doré et al. 1996; Lee et al. 2015; Macpherson and Roberts 2010). In other words, any positive result should lead to an extra round of testing subjects with the same paradigm but the addition of a distracting stimulus, which should cause a major breakdown in performance if the task indeed requires the involvement of WM.

Despite the various methodological challenges, cross-species comparisons of WM can provide valuable insights into key differences in cognitive abilities across taxa. WM may vary quantitatively rather than qualitatively among species. For example, humans can maintain 3–5 objects in their FoA, but are also able to almost double this number through exerting executive control over the contents of memory (Manrique et al. 2024; Read et al. 2022). In contrast, other species with lower cognitive capacities may access only one or two objects and may be less capable of combining information to form higher chunks (Cowan 2022; Cowan et al. 2024).

## The current study

We used the cleaner wrasse *Labroides dimidiatus* (hereafter “cleaners”) for our study as it constitutes a suitable model organism to explore the potential cognitive toolkit of ectotherms. Despite exhibiting an average brain-to-body size ratio for a fish (Chojnacka et al. 2015), cleaners possess a remarkable cognitive toolkit (Bshary and Triki 2022) that includes basic perspective-taking (McAuliffe et al. 2021), transitive inference (under certain circumstances, Bonin and Bshary 2023; Hotta et al. 2020), generalized rule learning (Wismer et al. 2016), chaining and configurational learning (Prat et al. 2022; Quiñones et al. 2020), true mirror self-recognition (Kohda et al. 2019, 2022, 2023) and reversal learning (Triki and Bshary 2021). Particularly relevant to our study is their ability to delay gratification, which is comparable to that of non-human primates (Aellen et al. 2021), despite an apparent lack of the general intelligence factor *g* (Aellen et al. 2022). Furthermore, cleaners remembered the *when* and the *what* after 2.5–15 min in a foraging task (Salwiczek and Bshary 2011). Although this may qualify as episodic-like memory (Manrique and Walker 2017; Pause et al. 2013; Tulving 1972, 2002), it raises the question about WM as cleaners seem to adjust and update their behavior flexibly in response to previously acquired knowledge. This prompted us to modify the experiment so that it would require WM.

To encompass several aspects of the definitions of WM, we designed experiments that varied in both the level of complexity and the specific context, aiming to provide a comprehensive preliminary assessment of WM in cleaners. We present the results from four experiments. These experiments involve, in part, adjustments from three others that led to inconclusive data (see supplementary material for additional methodologies and explanations for experimental designs that we abandoned). Importantly, all the presented experiments were supposed to incorporate additional tests, such as the sensitivity to disturbance and supplementary items to hold in WM, in order to assess all the pre-cited criteria of WM. The results made it unnecessary to pursue after the initial designs.

The first experiment, referred to as the “windows experiment”, involved placing food items inside Plexiglas rings (windows) attached to a Plexiglas plate, making the items invisible when the fish approached the location from the side. We also varied the ecological relevance of this test by using a plain white plate or a fish picture (considered relevant as mimicking natural cleaners—client interactions) to assess whether this influenced performance. Although this experiment was intended as a training step for a more complex one (see supplementary material), the data provided initial insights into the investigation of WM in cleaners.

The second experiment involved a movable arena where cleaners had to make several choices to access and eat all the hidden food items on a compartmentalized plate. In many regards, this was similar to the self-ordered search task for chimpanzees described in Völter et al. (2019) and adapted from Diamond et al. (1997) where individuals had to look for food rewards in different boxes while avoiding repeating a previously searched box. Solving this task relies either on remembering the spatial location that the boxes occupied in previous trials or on their exterior perceptual appearance. In our study, both Experiments 1 and 2 investigated WM with respect to the spatial distribution of food items.

In the remaining two experiments, we investigated the potential presence of WM in a visual context. In experiment 3, we used a methodology similar to Salwiczek and Bshary (2011), who challenged cleaners in simultaneous two-choice trials to remember which of two plates had been inspected 2.5–15 min earlier in order to choose the plate that would yield a food item on its backside. The process was very much a DMTS (or odd-one-out, depending on the condition): we first introduced one plate, letting subjects eat a single item on its back before removing the plate. We then reintroduced the same (now empty) plate alongside a second one that still had a food item available on its back. In experiment 4, we modified the methodology so that both plates were always visible to cleaners. This change in methodology addresses two potential issues with experiment 3: (i) the possibility that negative results in experiment 3 are due to subjects having misunderstood the task by interpreting the reintroduced plate as a new object, despite its identical color; and (ii) that a positive result is due to cleaners relying on familiarity alone (the presence of both plates from the beginning makes it less likely, although we note that the degree of interaction is still different).

To ensure that experiments 2–4 did not fall within the domain of sensory memory (Darwin et al. 1972; Sperling 1960), we set a minimum delay of 5 s between the potential encoding of information and its retrieval. We set a maximum delay of 10 s to limit the possibility that differences in information rehearsing abilities could overshadow differences in WM (Morey and Cowan 2018) and avoid measuring abilities strictly related to long-term memory (LTM), as previous research suggests that in humans, information can transfer into LTM within 15 to 18 s (Atkinson and Shiffrin 1968; WM and LTM discussed in Rhodes and Cowan 2018 for example). Delay times could be extended in future studies to investigate the influence of delay duration in greater detail.

Given the apparent lack of a general intelligence factor *g* in cleaners, we anticipated predominantly negative results, particularly in the more complex experiments. One of our main objectives was to assess whether the correlation between WM and *g* observed in other species holds for cleaners, with the absence of *g* implying limited WM capacity. However, cleaners frequently encounter multiple-choice scenarios in their natural environment that require the integration of previous knowledge into current decision-making. Thus, we also expected potential positive results in low-complexity experiments. As demonstrated in rats (Bratch et al. 2016), cleaners may possess different WM sub-processes, allowing for WM measurements across diverse paradigms.

## General methods

The study was conducted during two field trips in 2021 and 2022 to Mo’orea, French Polynesia.

We caught adult cleaners on non-protected reefs near Cook's Bay. We led cleaners into a barrier net (mesh size: 0.5 cm) using hand nets. Then, we placed them into zip-loc bags for transport and renewed the water after 45 min to ensure oxygenation. Fish were housed at the Gump Research Station in white opaque plastic aquaria, 70 cm (length) × 52 cm (width) × 45 cm (height), with a water height of around 30–35 cm. Seawater was pumped directly from the ocean into the flow-through system which was closed during experiments. Cleaners were first habituated to the aquaria and not tested until 8–15 days post-capture, during which time they learned to feed on plates and became accustomed to the basic material and experimenters. A camera placed on the experimenters’ foreheads recorded each trial.

All analyses were run with R software v. 4.3.1 (R Core Team 2022). For each model, we conducted an analysis of variance to compare two versions: one with the fish identity as a random factor for both the intercept and the slope with respect to time (trial number) and the other only with the fish identity as a random factor. We always kept the simpler versions as those were not significantly different, and they had slightly smaller AIC.

All datasets and R scripts are accessible on Figshare (https://figshare.com/projects/Cleaner_wrasse_failed_in_early_testing_stages_of_both_visual_and_spatial_working_memory_paradigms/231434).

The DRM of the French Polynesian government approved the fish collection and study design under authorization 11626/VP/DRM that covered 2021 and 2022. The fish adapted well to captivity and to human presence. They were released at their site of capture at the end of experimentations both years to minimize the environmental and population impacts of the research.

## Specific methods and results

For clarity, we present the methods and results together for each experiment.

### Experiment 1, windows experiment (spatial information-based paradigm)

#### Methods

**Fig. 1:**
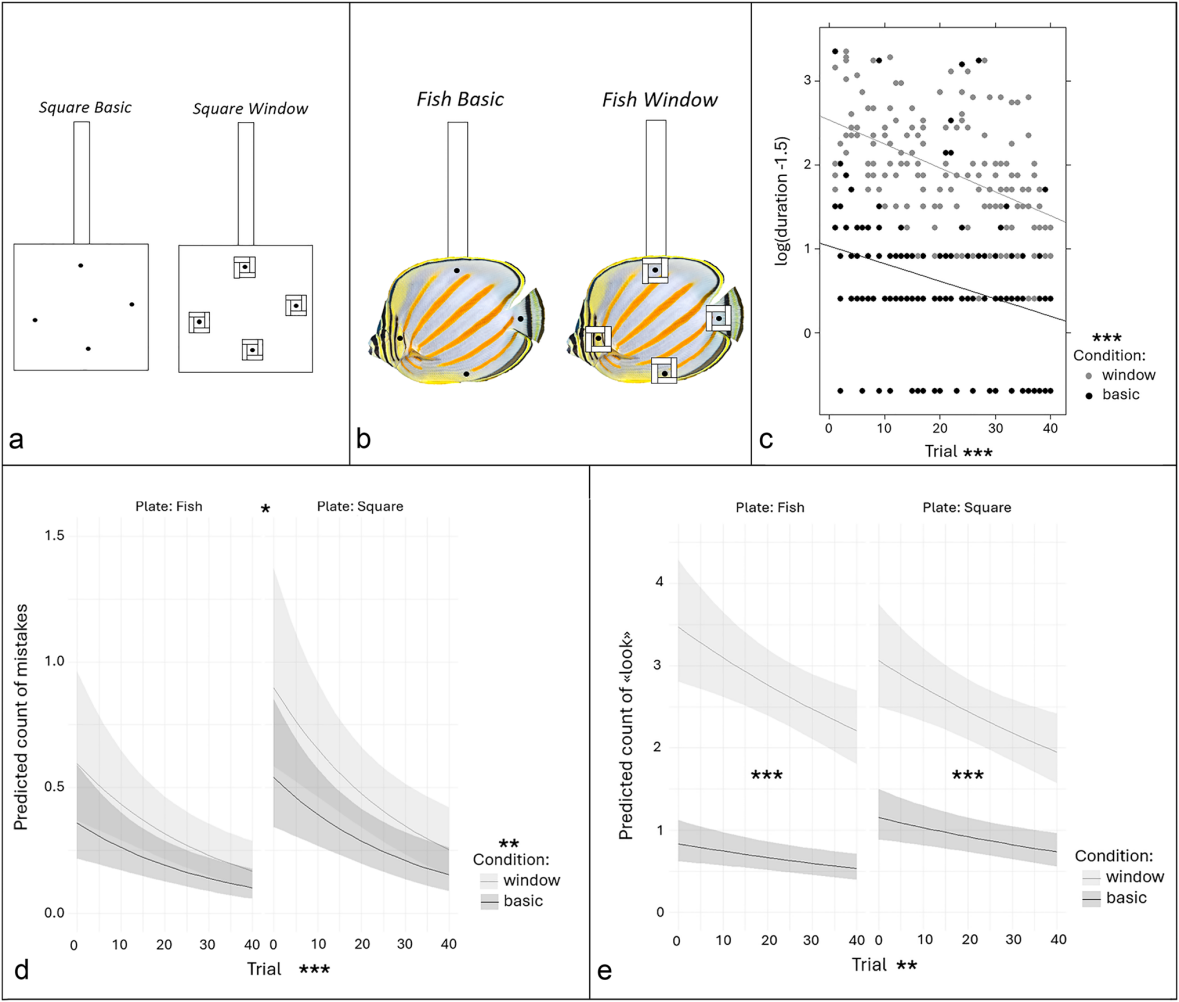
Windows experiments methods (**a**, **b**) and results (**c**–**e**). **a** White square plate and **b** butterflyfish plates. We used both types of plates in basic and window conditions. The black spots held mashed prawn items. Each window was 0.5 cm deep. **c** Effect of time and condition on the duration to eat the four items. The y-axis represents the log-transformed duration, and the x-axis shows the trial number. The color code indicates the condition. The stars indicate the significance of the associated variable. Significance codes: 0 ‘***’ 0.001 ‘**’ 0.01 ‘*’ 0.05 ‘.’ 0.1 ‘ ’ 1. **d** Effects of plate, condition, and time on the number of mistakes. The y-axis is the predicted mistakes count, and the x-axis displays the trial number. Each panel represents a plate, and the color indicates the condition. The stars indicate the significance of the associated variable. Significance codes: 0 ‘***’ 0.001 ‘**’ 0.01 ‘*’ 0.05 ‘.’ 0.1 ‘ ’ 1. **e** Effect of plate, condition, and time on the number of times cleaner looked around. The y-axis is the predicted count of times cleaners looked around (i.e., opportunities to see the items again), and the x-axis is the trial number. Each panel represents a plate, and the color code indicates the condition. The significance of the difference between both condition lines is given on each panel, and the significance of the covariate “Trial” is shown next to the x-axis label. Significance codes: 0 ‘***’ 0.001 ‘**’ 0.01 ‘*’ 0.05 ‘.’ 0.1 ‘ ’ 1

**Fig. 2:**
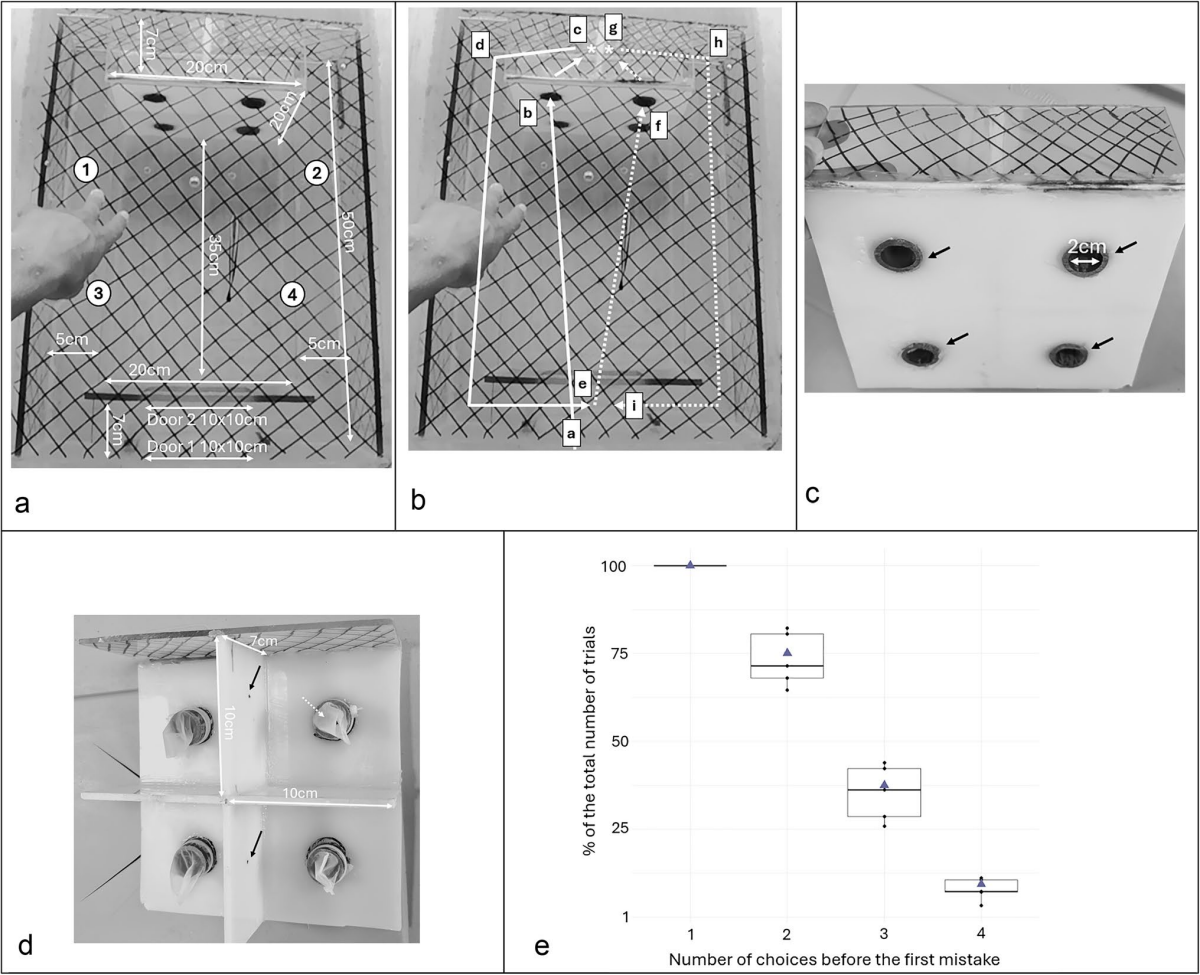
Working memory box, the movable testing arena. **a** Arena design. The main door, "Door 1," was manually operated by the experimenter, and cleaners entered the arena through this door to begin the trial. Swimming through the always open "Door 2" gave access to the central part of the arena and hence access to the plate with four pipes to access food behind. Corridors 1, 2, 3, and 4 lead from the respective compartments behind the plate back to the space between doors 1 and 2, allowing the fish to make a new run. Corridors 3 and 4 are positioned beneath 1 and 2, respectively. **b** Movements of cleaners in the arena. An example in which a cleaner first chooses to visit compartment 1 and then compartment 2. Letters on arrows show the sequence of movements. The cleaner eats food items (*) at stages c and g. **c** Front view of the compartment plate. Black arrows indicate the entrances to the four compartments. **d** Rear view of the compartment plate. A food item was placed on the internal wall of each compartment (black arrows), and the white-dashed arrow indicates the rubber curtain. **e** Percentages of the number of trials in which cleaners succeeded in making 1, 2, 3, or 4 correct choices in a row. The y-axis is the percentage of the number of trials, calculated on the usable trials only. The x-axis is the number of correct choices cleaners made before their first mistake. Because the first choice can never be a mistake, they succeeded in 100% of trials. Triangles indicate the random mean of success

Nine adult females, caught between 18.–20.9.21, were tested on four days from 7–10.10.21. These fish had previously taken part in abandoned experimental designs described in the supplemental material. We used two different plates with black spots marking the locations of four food items: (1) a plain white square (hereafter referred to as “square”, 20 cm × 20 cm, Fig. 1a) and (2) a laminated picture of a butterflyfish (*Chaetodon ornatissimus*, hereafter referred to as “fish”, 20 cm length × 14 cm height, Fig. 1b). Locations of items were up, right, down and left, which corresponded to specific areas on the fish plate (e.g., dorsal fin, gills, anal fin and caudal fin) to mimic natural ecologically meaningful body areas. We applied two conditions to both plates: (i) ‘basic’ where four food items are always visible, and (ii) ‘window’ where the four food items are placed inside Plexiglas wells of 1.5 × 1.5 cm and hence become invisible unless faced, Fig. 1a, b.

We conducted ten trials per fish per day for four consecutive days. Of the nine cleaners, four initially performed 20 trials using the fish plate, while five began with the square plate. The condition (basic or window) for each trial was counterbalanced, ensuring that no more than two consecutive trials featured the same condition. Each condition was tested five times per day.

Each trial followed the same procedure: the cleaner was placed behind a transparent barrier, with the plate inserted on the opposite side of the aquarium. Once the cleaner was released, we recorded the sequence in which it ate the food items, and counted the number of mistakes (i.e. the number of times it picked at an already-eaten location). Based on observations during data collection, we also extracted additional information from video footage, including: the total time taken to eat from all locations (if achieved, hereafter called “duration”), instances of “inspection” (i.e., the cleaner swimming around the plate, sometimes visually examining the locations without picking on the black spot), and instances of “leaving” (when the fish moved at least 5 cm away from the plate, marked by a line at the aquarium's base). Both inspection and leaving behaviors, which were grouped under the categorical variable “looks”, allowed the fish to re-examine (i.e. look again) the plate, potentially preventing mistakes. These three variables (number of mistakes, duration, and looks) were then used as three distinct response variables to estimate foraging efficiency. Plates were left in the aquarium for a maximum of one minute, but data were only extracted for the first 30 s, as eating the four items should occur fairly quickly.

#### Statistical analyses

We categorized our measurements into three response variables: the duration of time (maximum of 30 s) taken to eat the food items from all four locations, the number of mistakes (repeated location), and the number of “looks”. We aimed to assess the effects of ecological relevance (represented by the plate: square or fish), visual information (or complexity, represented by the condition: basic or window), and learning (examined through performance trends across trials). We began with the most complex model, including all possible interaction terms, and performed model selection to identify the best fit.

*Discarded trials* Trials in which subjects failed to eat items from all locations within 30 s were excluded from the analysis. Additionally, one trial on Day 2 (8th trial) was omitted due to a camera malfunction that prevented data extraction, and one trial for Fish E (Day 1, 10th trial) was excluded because the plate was removed prematurely, preventing access to all locations.

*Duration data* Despite applying various transformations to our duration data, the distribution remained problematic, with none yielding homogenous variances. The best-fitting model was a linear mixed-effects model applied to log-transformed duration data, with a transformation parameter of − 1.5. The explanatory variables included the plate, condition, and trial number, along with all interaction terms. The identity of cleaner subjects was treated as a random factor, and heteroscedasticity was modelled between the “basic square” condition and other combinations using the VarFunc class from the *nlme* package (Pinheiro et al. 2007) in R. We simplified the model by retaining only the plate, condition, and trial number as fixed effects.

Mistakes and looks data: As both response variables represented count data, we used generalized linear mixed-effects models with a Poisson distribution. The same methodological approach as used for the duration analysis was applied, leading to similarly simplified models.

Models diagnoses and post-hoc analyses: Model diagnostics were performed using the *DHARMa* package (Hartig 2022) in R, where we examined outliers and checked for model assumptions. We retained all outliers in our analyses as there was no valid justification for their removal. Overdispersion was checked when required. For post-hoc analyses, we employed least-square means methods from the *emmeans* package (Lenth 2023) in R.

#### Results

Among the 350 trials included in our analyses, cleaners did not eat at all locations after 30 s in 19 of them. Among these, two were in the basic condition vs. 17 in window condition, and nine were with the fish plate vs. 10 with the square plate. Also, these trials were spread among eight of the nine cleaners (leading to the removal of 1–4 trials from analyses, respectively).

Duration: With the full model (i.e., with all possible interactions), the condition:trial number was only marginally significant (Type II Wald Chisquare tests, condition:trial, Chisq = 2.92, df = 1, p-val = 0.09). We first simplified the model by keeping only that interaction along with the other covariates to see whether this tendency would be confirmed. As it came out non-significant (Type II Wald Chisquare tests, condition:trial: chisq = 2.22, df = 1, p-val = 0.14), we finally removed it as well and kept the simpler model with the three covariates only. As a result, we saw that cleaners took 2.55 more time to eat the four items in the windows compared to the basic condition (Least-square means, basic: predicted mean = 3.31 s, SE = 0.15 and windows: predicted mean = 8.45 s, SE = 0.54, significance from Type II Wald Chisquare tests, condition: chisq = 354.95, df = 1, *p* < 2e–16, Fig. 1c). Performance also improved over time; cleaners were almost twice as fast to eat the four items on trial 40 compared to the first trial (Least-square means, trial 1: predicted mean = 7.43 s, SE = 0.55 and trial 40: predicted mean = 3.69 s, SE = 0.20, significance from Type II Wald Chisquare tests, trial: chisq = 73.12, df = 1, p-val < 2e–16, Fig. 1c). In contrast, the plate type (fish or square) was only marginally significant (Type II Wald Chisquare tests, chisq = 2.77, df = 1, p-val = 0.096).

*Mistakes* None of the interaction terms were significant in the full model (Type II Wald Chisquare tests, chisq max = 2.65, p-val min = 0.10, all df = 1). After simplification, we found that cleaners made around 1.7 times fewer mistakes in the basic compared to the windows condition (Least-square means, basic: predicted mean = 0.23, SE = 0.04 and windows: predicted mean = 0.38, SE = 0.06, significance from Type II Wald Chisquare tests, condition: chisq = 6.84, df = 1, p-val = 0.009, Fig. 1d). Note that ‘mistakes’ include cases in which the fish had not eaten the entire prawn item, and hence ate a small leftover. This explains why we scored mistakes even in the condition in which everything was visible. There was also improvement over time; the number of mistakes was 3.4 times less at trial 40 compared to trial 1 (Least-square means, trial 1: predicted mean = 0.55, SE = 0.1 and trial 40: predicted mean = 0.16, SE = 0.04, significance from Type II Wald Chisquare tests, trial: chisq = 13.69, df = 1, p-val < 0.001, Fig. 1d). Finally, cleaners made 1.5 times more mistakes (i.e., repeating a location) in the square compared to the fish plate (Least-square means, square: predicted mean = 0.36, SE = 0.05 and fish: predicted mean = 0.24, SE = 0.04, significance from Type II Wald Chisquare tests, plate: chisq = 4.22, df = 1, p-val = 0.04, Fig. 1d).

*Looking* In the full model, only the interaction between plate and condition came out significant (Type II Wald Chisquare tests, chisq = 5.15, df = 1, p-val = 0.023). In the simplified model, the interaction between plate and condition remained significant (Type II Wald Chisquare tests, chisq = 5.10, df = 1, p-val = 0.02). The interaction exists because when tested with the fish plate, cleaners looked 4.2 times less in basic than in window condition, while the factor was only 2.6 in the square plate condition (fish: basic: predicted mean = 0.66, SE = 0.09 and windows: predicted mean = 2.73, SE = 0.20, z-ratio = − 9.70, p-val < 0.0001; square plate: basic: predicted mean = 0.91, SE = 0.10 and window: predicted mean = 2.41, SE = 0.18, z-ratio = − 7.21, p-val < 0.0001, Fig. 1e). Overall, performance improved over trials (Type II Wald Chisquare tests, trial: chisq = 9.04, df = 1, p-val = 0.003, Fig. 1e). In the first trial, cleaners looked 1.55 times more often compared to trial 40 (trial 1: predicted mean = 1.77, SE = 0.16 and trial 40: predicted mean = 1.77, SE = 0.11).

### Experiment 2, working memory box: the movable arena (spatial information-based paradigm)

#### Methods

We started the experiment with eight males who had been caught between 24.–28.1.22. Three males did not properly habituate to the experimental design, leaving us with five males. Trials took place between 3.–17.3.22. Subjects had been exposed to mirrors before, which should not have affected their performance in this experiment in any obvious way.

The experimental design represents a more sophisticated version of the first experiment, testing again the subjects’ ability to remember which food locations they have already visited. At the start of each trial, the cleaner entered the arena when the experimenter opened the first door (door 1, Fig. 2a) and the trial started when the fish passed the second door (door 2, Fig. 2a) which led to the center of the arena (35 cm length, 20 cm width, Fig. 2a). Swimming through, cleaners could access a square plate (20 cm × 20 cm) that had four round holes with pipes of 2 cm diameter and about 2.5 cm length in it (Fig. 2c, d) leading to individual, separated compartments (square of 10 cm × 10 cm and 7 cm deep, Fig. 2a). A piece of latex glove used as curtain prevented the fish from seeing what was behind the hole and, in most cases, prevented cleaners from coming out the compartment through the pipe. In each compartment, there was one mashed prawn item placed on the central side wall (Fig. 2d). Because of the rubber curtain, cleaners had to exit compartments by the individual attending corridors (total length 50 cm, height and width 5 cm, Fig. 2a) that led to the initial starting point. Only from there could cleaners swim again through the still-open door 2 and again choose between the four holes. We let cleaners repeat such cycles until they had eaten all four food items (details of the procedure provided in Fig. 2a–d). The distances were such that the time interval from consumption of a food item until the next decision was typically within the 5–10 s interval that we had aimed for as long as the fish swam normally. We did not quantify time within test trials, but trials in which subjects stopped swimming were not considered. A more detailed control of time delays was planned in case cleaners succeeded so that we could have ruled out the possibility of relying on other types of memory. Note that we drew black lines on the external walls of the arena, and the corridors were built using frosted Plexiglas allowing cleaners to perceive the presence of walls and light to penetrate the arena. The advantage of the current design over a radial maze is that subjects always come back to the same starting point between choices, making it unlikely for a simple swimming routine to explain a potential success (although one should still control for it in case of success).

We provided extensive training over 15 days to habituate the cleaners to the complexity of the experimental setup. The training was conducted in intermediary steps: (a) picking a food item placed in the center of a dark circle drawn on a white plate; (b) replacing the dark circle with a piece of pipe; (c) inserting the pipe through a plate to create an entrance, leading the cleaner through it with food as a reward; (d) adding a piece of latex glove to the prototype; (e) utilizing the final compartment plate with food items positioned at their final locations (preventing the cleaner from changing compartments from the rear). Several reward plates were used to guide the fish through the box, particularly through the corridors. When the cleaner exhibited fear of the setup, the box was left in the aquarium with reward plates scattered around to encourage inspection. This process was repeated until the cleaner swam comfortably and swiftly through the corridors and through the entire box.

Trials started after cleaners were habituated to additional elements of the experimental setup, such as swimming through doors, familiarizing themselves with different swimming routes within the box, and adapting to the box's movement (because we had only one box, we moved it in-and-out aquaria for each trial, which caused significant water displacement).

#### Data

Data collection was challenging. In some cases, trials could not be completed due to external events causing behavioral disturbances, or because cleaners spent too much time in the corridors, or because cleaners attempted to bypass the task by moving around the arena or entering the corridor from the wrong side to access food. Since we scored the number of correct choices made by a cleaner before the first mistake, when a trial had to be stopped after X choices, we still included it to see whether cleaners could make X choices or less before making the first mistake. For instance, if we stopped the trial after the third choice due to one of the above-cited events, this trial was considered for the count on 1, 2, or three choices, but not for 4 choices. Ultimately, data from five cleaners were analyzed, with a variable number of usable trials for each individual.

#### Statistical analyses

We compared the probability of correctly selecting 2, 3, or 4 compartments consecutively by chance with the actual performance of cleaners. If decisions are made by chance, the expected probabilities are as follows: 100% probability of first correct choice, 75% probability of two choices correct in a row is (3/4 compartments still contain food), 37.5% probability of three choices correct in a row is (75% × 50%), and 9.375% of four correct choices in a row (75% × 50% × 25%). For each cleaner, we made the sum of the first 2, first 3 and all 4 correct choices and incorrect choices over all its trials. We used a Wilcoxon signed-rank test to evaluate whether subjects consistently performed better than expected by chance.

#### Results

Cleaners failed to perform 2, 3, or 4 correct choices in a row above that expected by chance (Wilcoxon signed rank exact tests, 2 choices: V = 6 and p-val = 0.81, 3 choices: V = 5 and p-val = 0.63, 4 choices: V = 3 and p-val = 0.31, Fig. 2e). While the power of the test was very low, we note that observed means almost exactly match the predicted values, and not a single individual performed at noticeably higher levels (Fig. 2e).

### Experiment 3, two plates experiment (visual information-based paradigm)

#### Methods

18 adult females, caught between 18.–20.9.21, were tested over 13 days from 23.10.–4.11.21. Three females were excluded from the analysis as they did not habituate to the experimental procedure leaving us with 15 test subjects. We conducted 200 trials over 13 days. The number of trials per day varied between 5 and 20. Day 1 (trials 1–5) was training (methods described below as for day 3 onwards) and data were not analyzed. On day 2 (trials 6-15), we briefly tried a different method that we analyzed separately from the data collected during subsequent trials (see below).

**Fig. 3:**
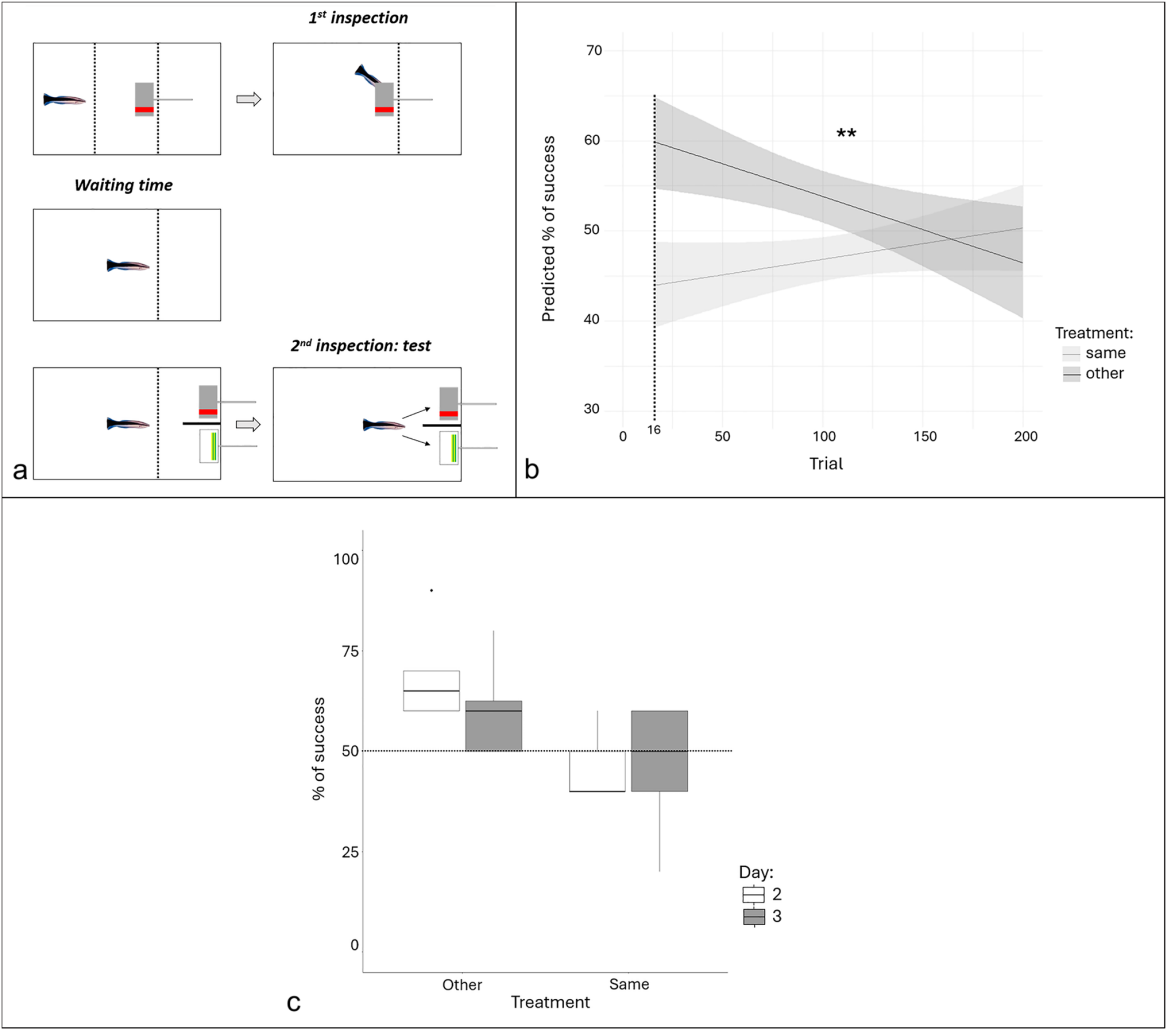
Two plates experiments. **a** The structure of a trial. Dotted lines represent see-through barriers. Each trial involved two inspections, with the cleaner expected to make a choice during the second inspection based on the plate presented during the first inspection and the assigned treatment. The waiting time between inspections ranged from 5 to 10 s. **b** Different effects of time on the proportion of success depending on the treatment. The y-axis is the predicted proportion (in percentages) of success, the x-axis shows the trial number, and the colors represent the treatments. Marginal effects plot showing the regression line and the 95% CI. In the “same” treatment, cleaners had to choose the plate that was the same as the one from the first inspection during the second inspection, while it was the other in the “other” treatment. The stars represent the significance of the interaction in the model. Significance codes: 0 ‘***’ 0.001 ‘**’ 0.01 ‘*’ 0.05 ‘.’ 0.1 ‘ ’ 1. **c** Percentage of correct choices. Boxplot showing the overall percentage of success (y-axis) of cleaners during the task, in both treatments (x-axis) and during day 2 and day 3. The dashed horizontal line indicates the random threshold of 50%

For the main data set (Trials 16–200), we divided the aquarium into three compartments using transparent barriers (Fig. 3a). Individual cleaners were initially located in one of the side compartments. We then allowed them to eat a food item (mashed prawn) from the back of one plate in the middle compartment. The plate was either grey with a red pattern on both sides, or white with a green and yellow pattern on both sides (Fig. 3a). The plate was then removed, and after a waiting period of 5 to 10 s, the cleaner was presented with both plate types on the other side compartment, so that one was identical to the initial plate. On day 2, we had the two plates placed and visible to the cleaners before we introduced the first plate in the middle compartment. While this facilitated the logistics for the experimenter, especially to stay within the 5 to 10 s time interval, this design was abandoned because the cleaner fish could obviously see the three plates and may have been less likely to consider the two identical plates as a unique one.

Half of the cleaners were assigned to the "same" treatment, where they would find a food item behind the identical plate, while the other half were assigned to the "other" treatment, where the food item was placed behind the different plate.

The plate presented during the first presentation was counterbalanced, and no more than two consecutive trials featured the same plate. During the actual trial, the position of the plates was also counterbalanced across trials. Cleaners were allowed to inspect both plates, regardless of their first choice, as this approach has proven effective in facilitating learning in associative learning tasks (R.B. pers. obs). We scored a trial as ‘correct’ if the cleaner approached the rewarding plate first.

#### Statistical analyses

The main statistical analysis uses data from trial 16–200. Data from day 2 (trials 6–15) were analyzed separately.

We performed a binomial generalized linear mixed-effects model, using the trial outcome (0: failure, 1: success) as the response variable, with treatment ("same" or "other"), trial number (from 16 to 200), and their interaction as explanatory variables. The identity of the cleaner was included as a random factor. Model assumptions were checked graphically using the diagnostic tools available in the *DHARMa* package (Hartig 2022) in R.

Post-hoc analyses were conducted using the least-square means method from the *emmeans* package (Lenth 2023) and simple slope effect calculations from the *reghelper* package (Hughes and Beiner 2023) in R. The same statistical methods were applied to analyze data from day 2 separately, allowing for comparison between days 2 and 3.

#### Results

We found a significant interaction between the treatment and time (Fig. 3b). In the “same” treatment, the success rate of cleaners increased by 6.3% over time, but this change was not statistically significant (Simple slope effect, slope = 1.001, t-value = 0.132). In the “other” treatment, the success rate decreased significantly over time by 13.4% (Simple slope effect, slope = 0.997, t-value = 0.008). Overall, both treatments resulted in success rates close but significantly different from the 50% expected by chance (Least-square means, same: predicted p of success = 47%, z-ratio = 2.41, p-val = 0.03 and other: predicted p of success = 53.6%, z-ratio = -2.4, p-val = 0.03).

During day 2 only, success rates were also significantly different between the two treatments (Least-square means, other: predicted p or success = 67.5%, SE = 0.052 and same: predicted p of success = 44.4%, SE = 0.052, z-ratio = 2.99, p-val = 0.003) but only the “other” treatment differed from chance (Least-square means, other: z-ratio = 3.06, p-val = 0.004 and same: z-ratio = -1.05, p-val = 0.293).

Success rates on day 2 versus day 3 were not significantly different (Type II Wald chisquare tests, day: chisq = 0.304, df = 1, p-val = 0.581 and treatment:day: chisq = 0.690, df = 1, p-val = 0.406, Fig. 3c).

### Experiment 4, dynamic two plates experiment (visual information-based paradigm)

#### Methods

23 cleaners (12 females, 11 males) caught between 22.1.–5.2.22. were tested in two groups, between 2.–15.3.22. Subjects were exposed to a foraging task before this experiment, so they were familiar with barriers and making choices at the onset of trials.

**Fig. 4:**
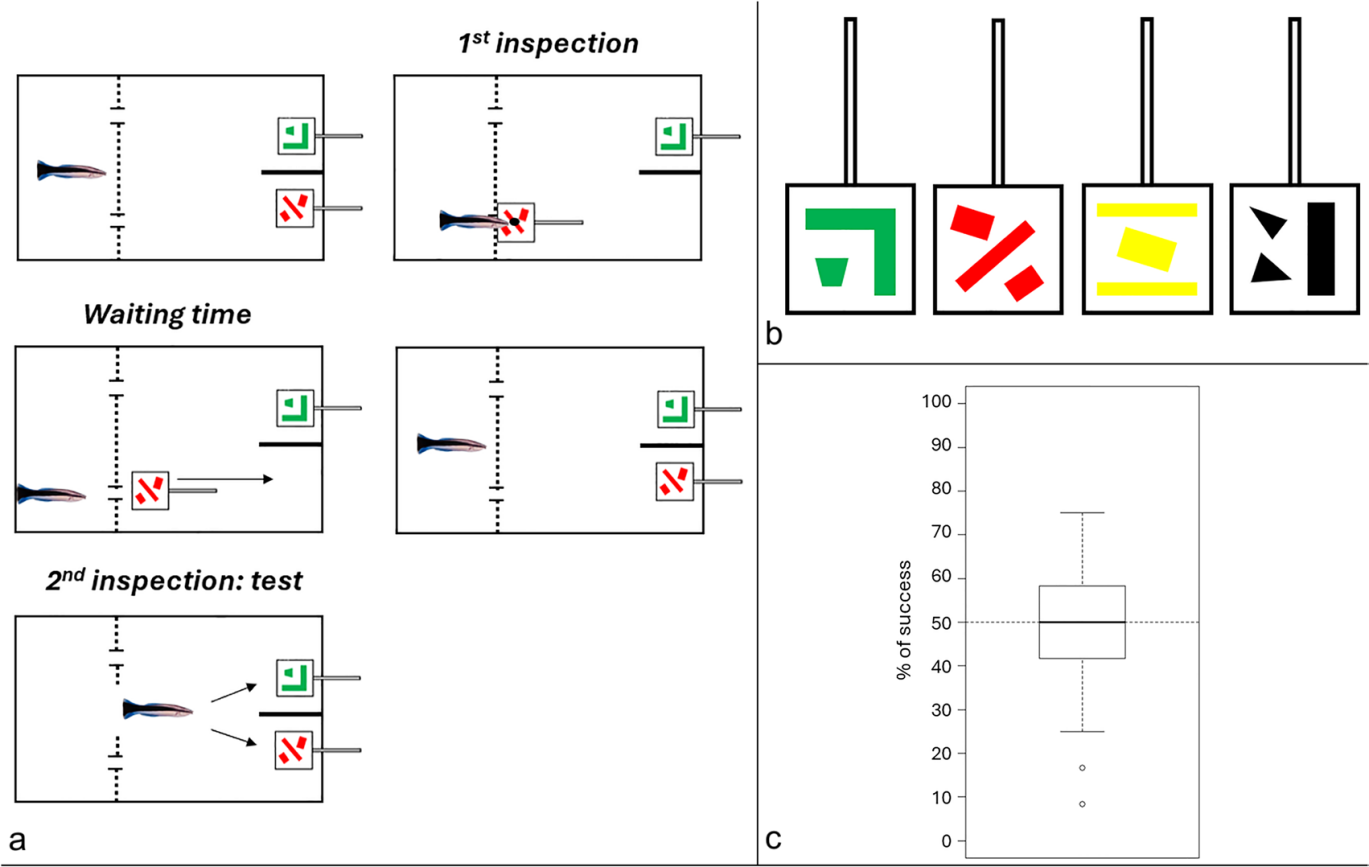
Experimental design. **a** Representation of a trial. Dotted lines represent the see-through barrier, and the interruptions indicate two holes. For each trial, we inserted the initial plate in the water, turned it around while moving toward the barrier to expose the food item hidden in the back (black spot). After allowing the cleaner to eat, we returned the plate to the back of the aquarium. Following a waiting period of under 10 s, the barrier door was opened, and we noted the choice of the cleaner. **b** The four different plates. Four different plates, each 5 cm × 5 cm with colored patterns (red, yellow, green, or black), were organized into six pairs. **c** Percentage of correct choices. Boxplot showing the overall percentage of success (y-axis) of cleaners during the task. The dashed horizontal line indicates the random threshold of 50%

This experiment was very similar in design to experiment 3. The main difference was that the first presented plate was not placed into the middle compartment and then taken out of the aquarium to be replaced by a look-alike. Instead, both plates were present from the beginning in the rear compartment. Then, during the first inspection, one plate was moved towards the middle compartment and turned such that the food item on its back became accessible to the cleaner by putting its mouth through a hole pierced in the Plexiglas see-through separation. The plate was then turned and placed back at its initial position. The door in the middle of the separation was then lifted, and the cleaner had to inspect the back of the other plate in order to obtain a second food item (see the steps illustrated in Fig. 4a). Note that there were not two treatments as in experiment 3, the correct plate was always the one that was different from the first inspection.

Another difference from experiment 3 was that we used four plates in total that differed in color and pattern (Fig. 4b). All six possible combinations were presented, each twice per day for a total of 12 trials per fish per day. We counterbalanced positions and which plate became accessible across trials. The idea behind using four plates was to increase complexity if cleaners solve the task with two plates by adding a third and eventually a fourth plate, and varying the number of plates that a cleaner gets access to before the barrier is lifted. As some cleaners did not participate in all trials attempted on Day 1 (eating through the hole appeared to be confusing at first), we only analyzed trials from days 2–4.

We measured the time interval between the end of the first plate inspection and the moment the barrier door was opened for the second inspection, aiming to stay within a 5–10 s interval. We noted the cleaner’s choice. If a cleaner selected the same plate as in the first inspection, the trial was considered a failure, and both plates were removed before the cleaner could access the food item on the other plate.

#### Statistical analyses

Trials where the time delay between inspections was greater than 10 s were not considered as those were outside the pre-established time limit.

To account for potentially confounding variables during data collection (e.g., the experimenter, the subject’s sex, the specific pair of plates and the trial number), we employed a binomial generalized linear mixed-effects model. The choice of the trial (0: failure, 1: success) was the response variable, and all confounding variables mentioned above were treated as covariates. The identity of the cleaners was included as a random factor. Since none of these variables was statistically significant, we used a null model to test the significance of the overall success rate.

For all models, we compared two versions using analysis of variance: one model with the cleaner’s identity as a random factor for both the intercept and slope with respect to time (trial number), and the other model with cleaners’ identity as a random factor for the intercept only. The simpler models were retained, as they showed no significant difference and had slightly lower AIC values.

We graphically checked the model assumptions using the diagnostic tools from the *DHARMa* package (Hartig 2022) in R. The significance of the variables was tested using Type II Wald Chi-square tests, and post-hoc analyses were conducted using the least-square means method from the *emmeans* package (Lenth 2023).

#### Results

Focusing only on trials in which the delay was between 5–10 s, we obtained a total of 1038 usable trials out of 1102 total (94.2 %). None of the covariates had a significant effect on the choice of cleaners (Type II Wald chisquare tests on binomial glmer, experimenter: chisq = 0.028, df = 1, p-val = 0.868; sex of fish: chisq = 0.117, df = 1, p-val = 0.733; pair: chisq = 3.034, df = 5, p-val = 0.695; trial: chisq = 0.504, df =1, p-val =0.478). The null model indicated that cleaners did not visit the correct plate above chance (Least-square means, predicted mean = 52.3%, SE = 0.016, z-ratio = 1.489, p-val = 0.136, Fig. 4c).

## Discussion and conclusion

This study comprises four experimental setups, some were novel, that may be indicative of or explicitly test for WM in cleaners. Overall, the performance of cleaners was modest. This was the case even for the two visual tasks that could, in principle, be solved without the recruitment of WM. For instance, familiarity, which is the ability to react to a stimulus without explicit recollection of the information associated with it, could have been used to solve Experiments 3 and 4 without the use of WM (the difference between familiarity and recollection is detailed and reviewed in Yonelinas 2002). In other words, an individual can be automatically attracted to an unknown (or unfamiliar) stimulus (or conversely, automatically avoid it) without the involvement of WM. Significant results were weak and inconsistent. For instance, in experiment 3, the significant interaction between treatment and the number of trials showed that the initial effect disappeared over time.

The absence of clear positive evidence for WM in any task made it unnecessary to conduct further testing aimed at ruling out alternative problem-solving mechanisms, like the development of a feeding sequence routine in experiment 1 and 2 (windows experiment and WM box) or the use of familiarity in experiment 3 and 4 (two plates and dynamic two plates experiments). Similarly, the lack of positive evidence also made it unnecessary to test for sensitivity to disturbance or storage capacity of WM (e.g., number of items that can be held and manipulated simultaneously), two key aspects associated with WM capacity (Table 1) and largely overlooked in the literature. We recognize that the absence of evidence cannot be perfect proof for evidence of absence, but consistent results across the four different experiments make us sceptical about the existence of WM in cleaners. Previous laboratory experiments with cleaners highlighted the use of strategies that capitalized on previous knowledge and experiences (Binning et al. 2017; Bshary and Grutter 2006; Grutter 2004; Pinto et al. 2011). Because the deployment of such strategies seems to require agile behavioral adjustments, relying on executive control and adaptability, we had expected WM to be recruited (Baddeley and Hitch 2019; Cowan 1995; Diamond 2013). We provide detailed discussions of each experiment in the supplementary material, including details of earlier setups that were confusing for cleaners and hence, were not fully carried out.

It is also important to keep in mind that, as introduced above, if cleaners had succeeded in the paradigms presented in this study, further analyses and experiments would have been required. For instance, one way to succeed in the windows experiment and the WM box could have been to develop a routine, i.e. a systematic order in which to eat the items. In the windows experiment, always swimming in a circular path around the plate would have led to high performance independently of the presence/absence of the windows. Although establishing a routine order would have been more difficult in the WM box, as each choice had to be made at a location equidistant from all possible locations, we would still have had to check for such routines if individuals had performed above chance levels. To prevent the use of routines, an ideal next step in the design would have been to initially block access to certain options and vary which options were blocked between trials. In our two-plate visual paradigms (two plates and dynamic two plates experiments), success could have been explained with a familiarity rule, i.e., subjects learning to seek or avoid the familiar stimulus, that is, the first plate. While this familiarity issue is less pronounced in the dynamic two plates experiment (as both plates are always present, though the degree of interaction remains different), positive results in any of the four paradigms would have required testing how a disturbance affects performance. Thus, the fact that cleaners produced negative results shows not only that they did not use WM, but also that they did not rely on any simpler mechanisms that could have yielded above-chance performances, such as the development of a routine or use of familiarity. This in itself could be the object of future studies.

## Link to ecology

We strived to design experiments that could reflect the ecology of cleaners, with the goal of engaging their WM abilities, if such abilities exist. For example, when cleaners clean their clients, they inspect different surface locations (e.g., mouth, gills, tail). For the sake of efficiency, it would be expected that cleaners remember and hence, avoid revisiting previously inspected locations (i.e., they optimize their foraging strategy). Avoiding previous locations should reflect the ability to store and update information, a key signature of WM. Our “windows experiment” and the “movable arena experiment” aimed to elicit this ability on a single unit offering food items, as a client in nature. Indeed, the cleaners’ natural foraging behavior guided the development of our experimental designs, rather than using a radial maze that has no ecological relevance for cleaners. We found that cleaners were more efficient when windows were overlaid on an image of a client fish (here, a butterflyfish) *versus* overlaid on a plain white square plate. The picture of a client fish was intended to give additional ecological relevance to the experimental setup. While we found significant effects or a tendency that plate design matters, we currently cannot rule out that simple differences in coloring (rather than fish *versus* plate) may have affected the behavior of cleaners. In any case, the more important observation was that the presence of windows strongly reduces the cleaners’ foraging efficiency, independently of plate type.

The other two experiments, where we used pairs of plates, were also intended to simulate cleaners' ecology. Because cleaners have numerous interactions per day (Grutter, 1996), there are regularly two or more clients at a cleaning station (Bshary 2001). Due to such situations, we hypothesized that cleaners would likely remember the most recently inspected clients to avoid inspecting the same individual again, thereby optimizing their foraging behavior. Indeed, within a time window indicative of long-term memory, a previous study found that cleaners could adapt their foraging choices based on information they acquired several minutes before, avoiding “client plates” that they had inspected within 2.5 to 15 min time windows set by the experimenter (Salwiczek and Bshary 2011). In other words, cleaners could remember "*when* they interacted with *what* after a single event" (Salwiczek and Bshary 2011). Although the possession of something akin to episodic-like memory would account for these results (Pause et al. 2013; Tulving 2002), it seemed reasonable to expect that their ecology selects cleaners to succeed also when the time intervals separating acquisition and retrieval are shorter, which would more likely require WM. Lack of success in this new condition reinforces the idea that time is a critical factor for eliciting WM and that the separation between LTM and WM is a valid one, even if one is to accept that WM could operate with information reactivated from LTM (Cowan 2008, 2019; Norris 2017).

The apparent lack of WM in cleaners highlighted by our four experiments forces us to reconsider the idea that WM would be recruited to optimize foraging strategies. Instead, cleaners might use various simpler mechanisms to still be rather efficient foragers. In the context of a single interaction, cleaners might optimize their food intake by applying learned routines or by simply starting with the most inspected body part before searching more randomly. When multiple clients are present at a cleaning station, the most important task for foraging efficiency might be selecting the most profitable client, which involves identifying species and applying the corresponding behavioral rules (Triki et al. 2019), an ability that does not require WM. For individuals who do not leave the cleaning station immediately, behavioral reading (e.g., identifying a reduced interest in a cleaning interaction) may allow cleaners to make appropriate choices without relying on WM. However, in most cases, clients leave after the cleaner terminates the interaction, allowing cleaners to simply swim toward an approaching or waiting client. The fact that cleaners can solve a similar task after a longer time interval between exposure and test (2.5 to 15 min; Salwiczek and Bshary 2011) but fail in our paradigms suggests that in the wild, such decisions might typically occur after a longer delay. Clients might return more frequently after few to several minutes rather than just a few seconds. Lastly, as pointed out before, a familiarity mechanism (Yonelinas 2002) might cause an automatic attraction of cleaners to newly arriving clients.

A peculiarity of cleaners is that they have 800–3000 cleaning interactions per day (Triki et al. 2018), providing them with an extraordinary number of learning opportunities. According to the CON framework (Bonin and Bshary 2025), ecological needs (N) can be met through a combination of cognitive tools (C) and learning opportunities (O), where high C or high O may compensate for low O or low C, respectively. In the cleaner wrasse case, vast learning opportunities may compensate for a lack of complex cognitive abilities—such as WM—to succeed in their daily challenges (Bonin and Bshary 2025).

## The lack of WM in cleaners, and other ectotherms?

We did not anticipate that cleaners would perform so poorly in all four experimental tasks. However, the lack of positive evidence for WM fits the apparent absence of a general intelligence factor *g* reported for this species (Aellen et al. 2022). In mammals, individual performance in WM tasks is a reliable predictor of performance in multiple other cognitive tasks, indicating that the presence of WM is a precondition for the presence of the *g* factor (Ackerman et al. 2002; discussed and reviewed in Conway et al. 2003; Engle et al. 1999; Shipstead et al. 2014). Consequently, we did not expect that the performance of cleaners would be comparable to that of large-brained endotherm species (e.g., primates or corvids). Another potential difference in WM abilities between ectotherms and endotherms could be related to the sensitivity to disturbance, with cleaners being possibly more sensitive compared to other species (discussed in Carruthers 2013 and Manrique and Walker 2017). This hypothesis needs specific testing using paradigms where the fish show initial success in the absence of disturbance. Nevertheless, a lack of any significant evidence for WM using ecologically relevant low-complexity experiments in cleaners came as a surprise. As it stands, differences among species in WM capacities can, in principle, be quantitative, as for example between humans and chimpanzees (Manrique et al. 2024). Our experiments 2 and 4 were designed such that cleaners could have performed at a low level (above chance for the second choice in the movable arena experiment, above chance as long as only two plates are present in the dynamic two plates experiment) but not when the required information increased (like for the 3rd and 4th choice in the movable arena experiment, or when confronted with 3 or more choices in the dynamic two plates experiment). Success with a single choice being indicative of a WM of size 1 (i.e., with one stored item), we had expected that such a small capacity would be present in this species given their proficient problem solving, yet data did not match our expectation.

One more avenue should be explored where negative results would further strengthen our confidence that cleaners lack WM. Research on New World monkeys has revealed that apparently challenging two-choice tasks (on gaze following and prolonged object permanence) lead individuals to readily accept the 50% probability of receiving a reward purely by chance (Burkart and Heschl 2006; Schubiger et al. 2016). However, when the chance probability was reduced to 11-13%, individuals paid more attention and subsequently performed above chance (Burkart and Heschl 2006; Schubiger et al. 2016). Since WM tasks demand attention, more complex versions with lower probabilities of chance success could potentially enhance performance, provided that WM is present in cleaners. Nevertheless, a larger question arising from our study is whether the apparent absence of WM could be specific to cleaners, or whether it could represent a fundamental cognitive difference between ectotherms and endotherms (Jerison 1969). The consensus in the literature is that WM is a system that keeps recently acquired information in an active state, allowing its combination with knowledge previously acquired, stored in the long-term memory and reactivated, to assist prospective adaptive action (e.g., Baddeley and Hitch 1974; Cowan 1995, 2019). With this definition in mind, we consider the extant literature regarding WM in fish to be very incomplete (Table 2). Studies to-date have investigated the natural alternation rate by zebrafish in a Y-maze during a free exploration task (Fontana et al. 2021) and have showed that zebrafish succeed in a delayed match-to-sample experiment as long as the delay did not exceed 4 s (Bloch et al. 2019). Furthermore, both cichlids and guppies selected for a bigger telencephalon size showed significant, though modest, success in the Piagetian stage 4 of object permanence (i.e., recovering a hidden object, Piaget and Cook 1952), which the authors directly associated with WM (Guadagno and Triki 2024; Triki et al. 2023). These are all interesting results that can be compared to the performance of endotherms. However, these paradigms may not test for WM. Maze studies to measure WM have been criticized for not considering whether the time interval separation between acquisition and retrieval is within a range compatible with WM (Carruthers 2013). In addition, the manipulative and problem-solving aspect of WM is barely detectable in the task, especially when it is about purely explorative behavior, as in Fontana et al. (2021). Match-to-sample tasks are visual tasks, often considered "recognition tasks" (Bachevalier and Mishkin 1986; Zola et al. 2000), where processes like familiarity could satisfactorily account for the observed positive results, without invoking higher-level executive control (e.g. Aggleton et al. 1986; Cowan 2019; Yonelinas 2002), especially if the tested delay are within the range of non-manipulative short-lived storages (Table 1) (Bloch et al. 2019). Regarding experiments on object permanence, we argue that only later stages (i.e., Piagetian stages 5 and 6) can reliably be linked to WM, as the combination and manipulation of information are absent in previous stages. In Piagetian stage 4 (single visible displacement task, Piaget and Cook 1952), the subject must only retrieve a hidden object. Therefore, we would argue that there is no necessity for the combination or manipulation of previously acquired information. In contrast, later stages (5 and 6, with invisible displacement, Piaget and Cook 1952) almost certainly engage WM, as they require varying degrees of mental computation to track the trajectory of an unseen moving object.

**Table 2:** Previous experiments on ectotherms in which authors linked performance to the operation of WM

Type	Task	Species	Authors’ conclusions	Potential limitations	References
Spatial	FMP Y-maze; look at spontaneous arms alternation; 15 min free exploration recording	Zebrafish	Modification of spontaneous alternation following drugs administration indicates an enhanced WM	No manipulative aspect, no disturbance test, time interval not controlled	Fontana et al. (2021)
Spatial*	Object permanence (Piagetian single visible displacement, less than 10 s)	Guppies	Success at 60% linked to bigger telencephalon size, guppies have “at least substantial WM” (p. 6)	No manipulative aspect, no disturbance test	Triki et al. (2023)
Visual	Delay matching-to-sample (DMTS), 1 to 12 s	Honeybees	WM in bees is “robust, and yet plastic” (p. 1) Subjects succeeded above chance until around 9sec (around 55% of success between around 5 and 7 s)	No disturbance test, familiarity as an alternative explanation	Zhang et al. (2005)
Visual	DMTS (3 to 5 s)	Zebrafish	Zebrafish have WM, but success is limited to certain conditions (training, type of response). Subjects always failed with a delay greater than 4 s	No disturbance test, familiarity as an alternative explanation	Bloch et al. (2019)
Visual	Expectancy-violation experiments**, 90sec + max 300 s	Jumping spiders	Objects can be held and manipulated (here, prey orientation) in WM	No disturbance test, familiarity as an alternative explanation, long time interval	Cross and Jackson (2014)

We provide information on the general type of task, study species, the conclusions by the authors, and the potential limitations with the methods, i.e. what critical features of WM according to our definition are not part of the experimental design. We focused on research that occurred after 2000, notably due to the constant evolution of WM models. Thus, the table does not present a full review of the literature on the subject

*We indicate “spatial” because the object is hidden at a location that the tested individual must inspect. However, the concept differs from a standard spatial paradigm in that it is also linked to the existence of the object itself

**Design incorporating match-to-sample (determining whether the prey was similar or different from the one seen at the beginning) and object permanence (the prey is hidden and later revealed– either the same one or a different one)

Thus, to answer the question as to whether or not WM is present in ectotherm vertebrates, we need to conduct experiments that are explicitly designed to test for this executive function. Importantly, we are confident that performance above chance in experiments 3 and 4, in combination with a lowered performance when a disturbance before the choice is introduced, would provide fairly conclusive evidence for the presence of WM (although the problem-solving level remains fairly low) (Carruthers 2013; Cowan et al. 2024). These paradigms can be applied to other species, allowing for cross-species comparisons.

In conclusion, our comprehensive series of experiments yields no evidence for WM abilities in cleaners, which contrasts with evidence for WM in endotherm vertebrates, including corvids and psittacids (Pepperberg and Funk 1990; Zucca et al. 2007), mice (Kolata et al. 2005), rats (Bratch et al. 2016) and chimpanzees (e.g., Völter et al. 2019, debates in Manrique et al. 2024; Read et al. 2022). This contrasts with research on the other two main executive functions (EFs)—cognitive flexibility (Aellen et al. 2022; Parker et al. 2012) and inhibitory control (Aellen et al. 2021; Lucon-Xiccato and Bisazza 2017; Sovrano et al. 2018)—for which evidence has been found in fishes, including cleaner wrasse (Aellen et al. 2022). Taken together, the results raise the intriguing possibility that rather than all executive functions, WM and the highly associated *g* factor warrant an enlarged and reorganized brain as found in endotherms but not in ectotherms (Jerison 1969).

## Supplementary Information

Below is the link to the electronic supplementary material.

Supplementary file1 (DOCX 362 kb)

## Data Availability

All datasets and R scripts are accessible on Figshare (https://figshare.com/projects/Cleaner_wrasse_failed_in_early_testing_stages_of_both_visual_and_spatial_working_memory_paradigms/231434).
