# Supplementary material for: Cleaner wrasse failed in early testing stages of both visual and spatial working memory paradigms
Source: Anim Cogn. 2025 Jun 4;28(1):43. doi: 10.1007/s10071-025-01959-w (PMC12137418; doi:10.1007/s10071-025-01959-w)
Supplement: Supplementary file 1 — Supplementary file1 (DOCX 362 kb) [file 10071_2025_1959_MOESM1_ESM.docx]

Supplementary material

S.1. Methods

This supplementary document provides a detailed account of the various experimental designs we initially developed to assess WM abilities in cleaners. Throughout the testing phase, we faced several challenges, which prompted us to adapt our methods. Here, we describe both the intellectual approach and the methodological adjustments that were made, ensuring that readers gain a comprehensive understanding of how these factors shaped the experiments presented in the main text. All the designs discussed here were implemented during the fieldwork conducted in 2021 in Moorea, French Polynesia.

**S.1.1. The compartmentalized plate**

| 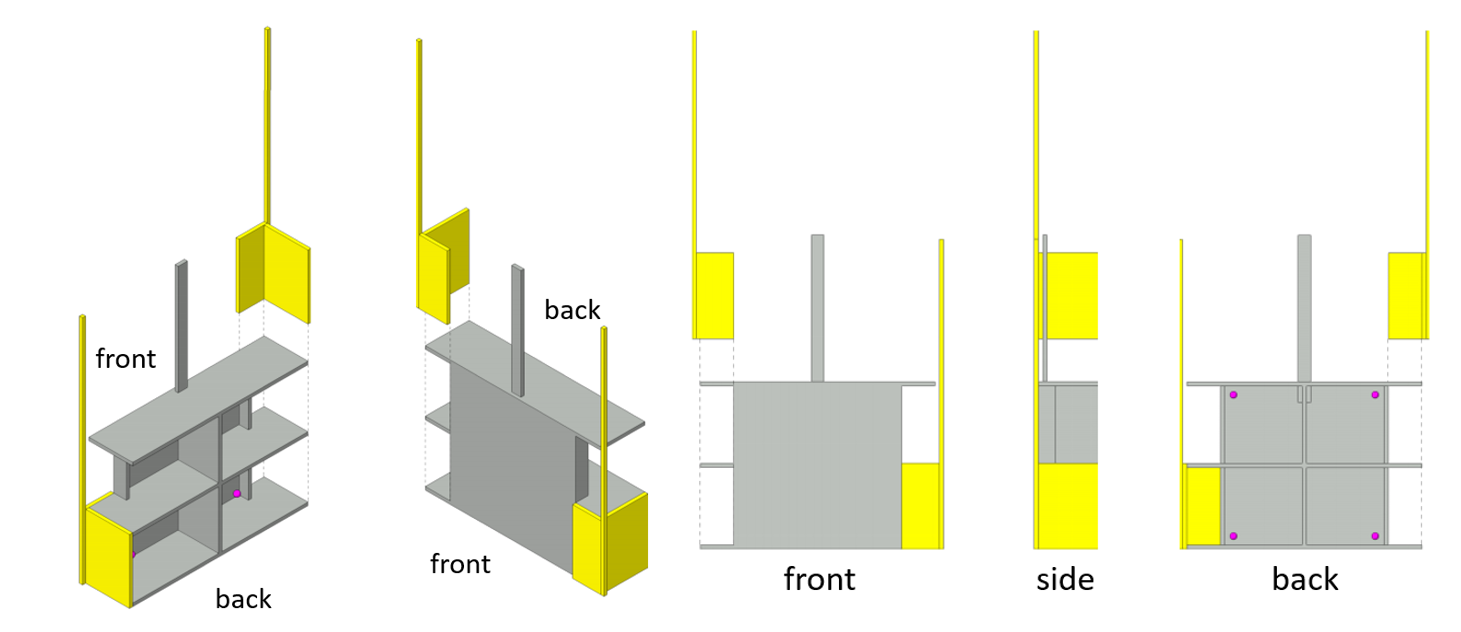 |
| --- |
| **Figure S1: The compartmentalized plate**  The compartmentalized plate (20 cm x 20 cm) consisted of four equal-sized compartments. Constructed entirely from white Plexiglas. A food item (represented by a pink ball) was placed in each compartment. We placed the plate with its back part against it to force cleaners to swim around to the front to change compartments. The doors (yellow structures) allowed us to selectively open or close specific compartments. We planned to use several variations of the plate design: one without colors (as shown here), another with each compartment marked by a different color, and a fish-shaped plate. |

The compartmentalized plate was the first design we conceived. We conducted the training in two phases to acclimate the cleaners to this setup (Fig. S1). First, we randomly placed the compartmentalized plate into the tank, with food items in all four compartments, allowing the wrasse to explore and habituate to it without exhibiting stress. Second, we closed the back of the compartmentalized plate, requiring cleaners to enter from the sides, which was intended as the final experimental condition.

We encountered several issues, some of which were material-based and fixable, such as needing to adjust the plate multiple times to ensure the wrasse could only access the compartments from the sides. However, other problems were more critical, as they involved cleaners’ behavior. For example, we observed that cleaners tended to remain inside the first visited compartment, which we initially suspected could be related to stress. However, their overall behavior did not support this idea (normal swimming when outside the compartment).

While cleaners occasionally moved to the adjacent compartment directly above or below, they rarely switched to the opposite side. As a result, we concluded that this setup was unsuitable for addressing our research question and decided to transition to a smaller plate design to reduce the stress and confusion.

**S.1.2. “First attempt”**

To address the limitations of the compartmentalized plate, we developed a new setup (Fig. S2) designed to test the cleaners under various conditions (i.e., complexities, Fig. S2a) and contexts (i.e., plate types, Fig. S2b). The idea was to present the cleaner with the different plate-condition combinations and observe their inspection behavior. We counted repeated visits to the same location as errors and aimed to assess whether condition and/or context influenced their success in eating all items (as in the “windows experiment” presented in the main text).

| 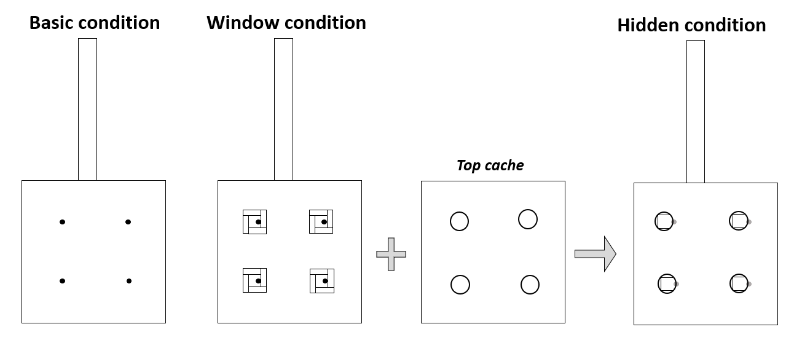  a | 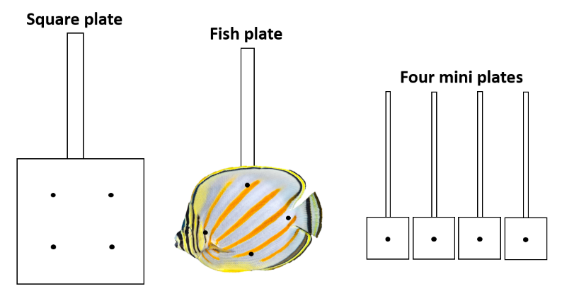  b |
| --- | --- |
| 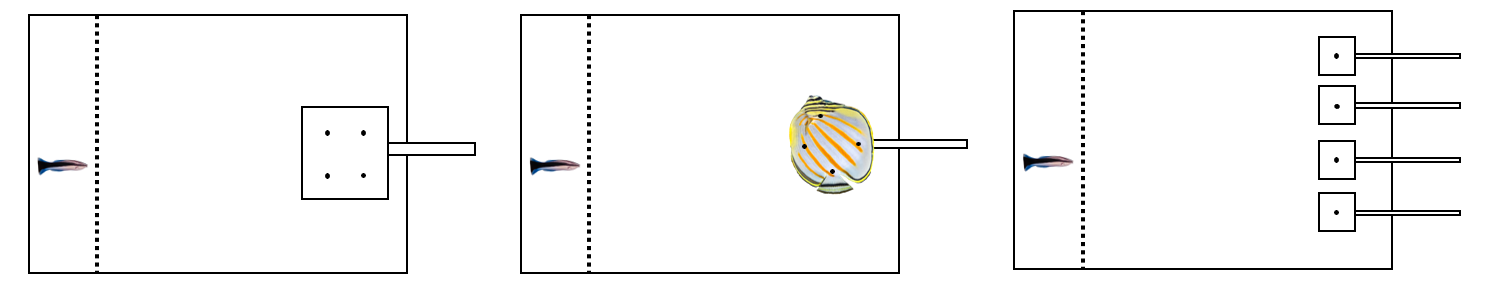  c | |
| **Figure S2: “First attempt” design.**  We used different experimental conditions (a) and plates (b), all with a similar setup (c). Food items were placed on designated black spots. There were three different conditions, each representing a different level of complexity (a): basic (items placed directly on black spots, the simplest), window (items placed within a shallow 0.5 cm Plexiglas window, moderate complexity), and hidden (a Plexiglas cover with holes added over the window plate, concealing the items, the most complex). We planned to test these using three different plate designs (b): a square plate, a fish-shaped plate, and four smaller plates. The experimental procedure involved placing the plate in the tank while the cleaner observed from behind a transparent barrier (c). Once released, the fish was allowed to explore the plate, with repeated visits to the same location being counted as mistakes. The trial ended when all locations had been visited. | |

In the basic condition, all items were visible, and we expected no errors from cleaners. In the "window" condition, items were hidden when cleaners approached from the side, meaning that any mistakes could suggest a lack of WM, as they would need to recall previous locations. Lastly, the "hidden" condition increased the complexity further, as the food item was entirely hidden. If the cleaner made no mistake here, we would check for routine-based behavior (e.g., inspecting locations in the same sequence every time). If no such routines were found, it would suggest that cleaners indeed possess WM.

We conducted five trials per cleaner but encountered an issue with the hidden condition: the cleaner either could not access the reward or could see it, thus violating the goal of the setup.

**S.1.3. “Holes plates”**

To overcome the issues encountered with the hidden condition in the previous design, we modified the setup by enlarging the holes on the cache to make it easier for the cleaner to access the food (Fig. S3a-b). To avoid the food being visible, we used rubber bands around each hole and placed the food reward behind them, requiring the cleaner to actively enter the holes. We also re-arranged the food items to mimic the layout of the fish plate.

The concept of this design was to allow the cleaner to inspect two locations and eat the food items during a first round of inspection (Fig S3b-d). Afterwards, we separated the cleaner from the plate, opened the remaining locations, and observed which ones it inspected in a second round (all locations were then available). Any repeated visit to the same location counted as a mistake. By randomizing the accessible locations during the first inspection, we minimized the possibility of the cleaner to rely solely on routine behaviors.

Two methods were tested during the inspections (Fig. S3c-d). The first method (Fig. S3c) was problematic due to delays between the last food item eaten and the second inspection. We aimed for a 5-10 second interval but struggled with separating the cleaner quickly enough without causing stress. Without this flaw, any negative results would have been reliable since cleaners always had visual access to the plate. The second method (Fig. S3d) streamlined the process, but we still couldn't consistently meet the desired time interval without stressing the fish, and some cleaners managed to see the food items, biasing the setup.

An additional simplified attempt involved closing three locations during the first inspection and opening just one during the second. However, manipulation challenges persisted, and we abandoned the setup.

Ultimately, we opted to return to a simpler design, comparing a “basic” and “window” condition from the first attempt setup (Fig. S2), as we thought it would already be a starting point. This led us to the "windows experiment," as described in the main study, followed by the "two plates experiment”, a design better suited for cleaners.

| 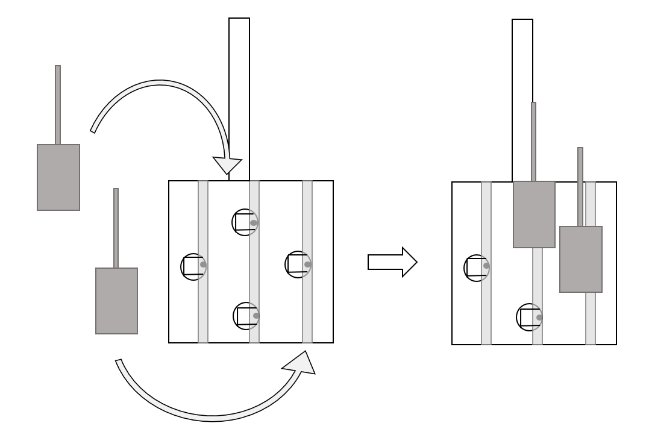  a | 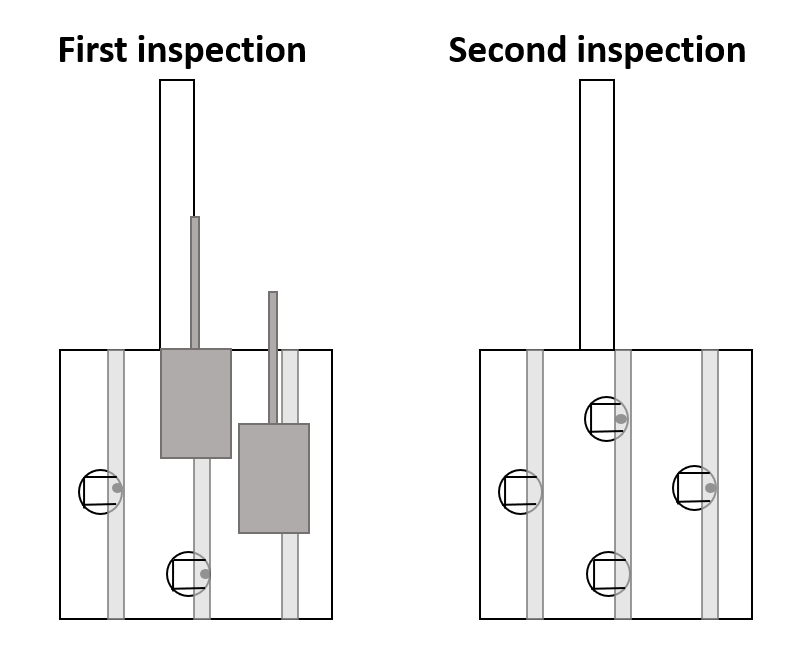  b |
| --- | --- |
| **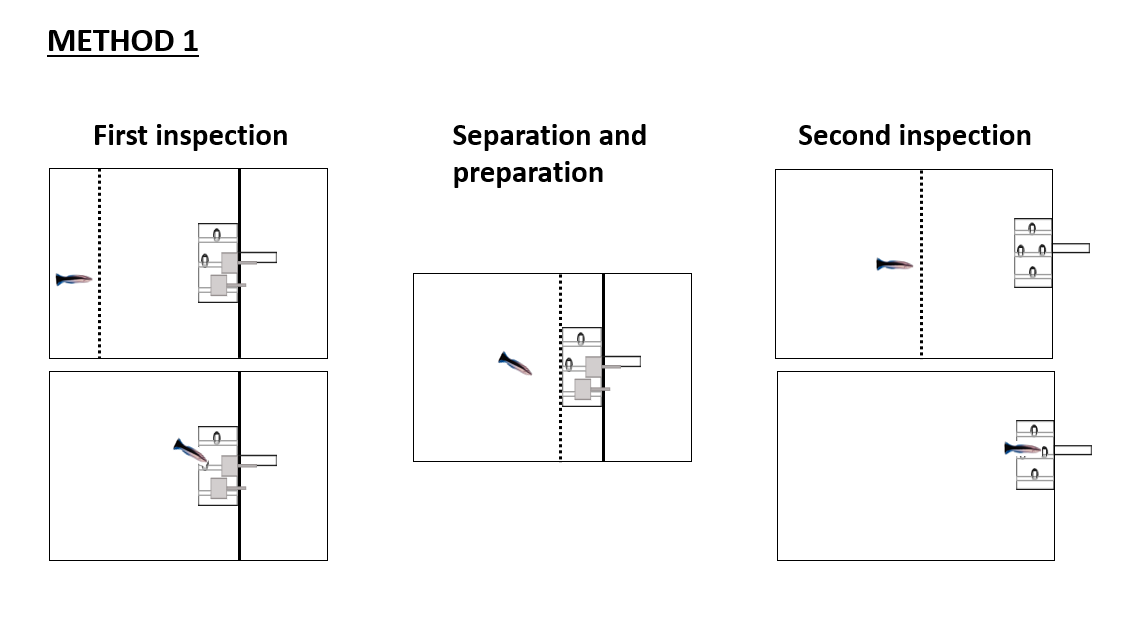**  c | |
| 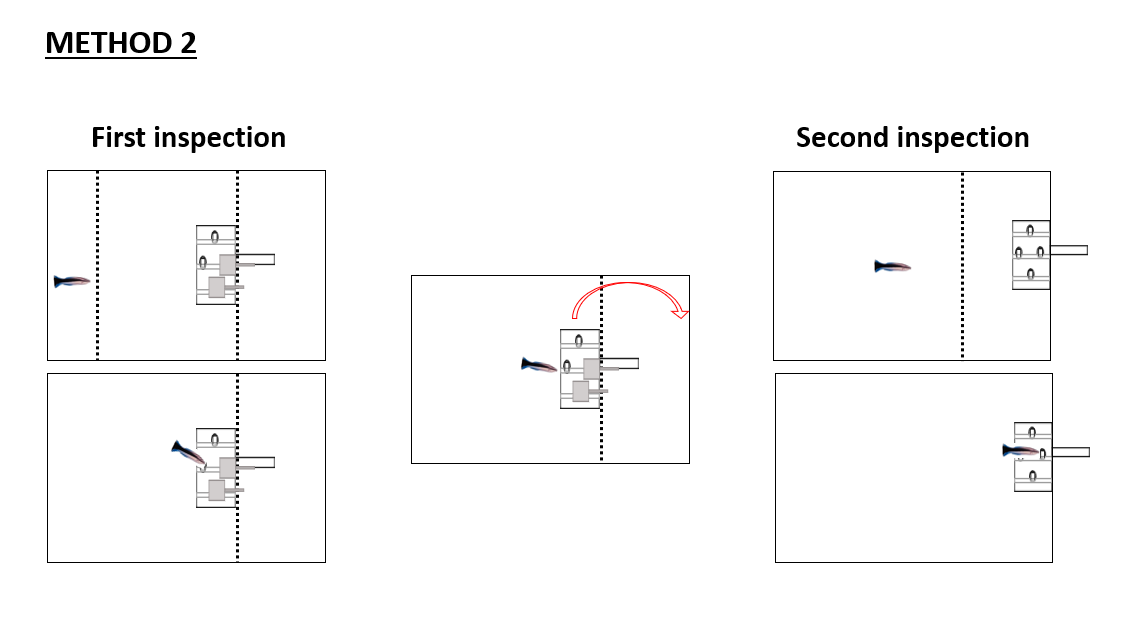  d | |
| **Figure S3: The “holes plate” design.**  The holes plate (a) and how we intended to use the different inspections (b). The plate was built using grey caches to block selected locations (a-b), and food items were placed on black spots behind rubber bands (clear grey vertical bands, a-b).  Two methods were tested to organize the inspections (c-d). For the first method (c), cleaners were allowed to feed at two locations during the first inspection. Afterward, they were gently separated from the plate using a transparent barrier (dotted line) and the plate was moved to the back of the aquarium before the cleaner was released for the second inspection. In the second method (d), aimed at reducing manipulation time, instead of removing the cleaner from the plate between inspections, the plate itself was moved to another section of the aquarium to facilitate the process. | |

S.2. Discussion

In this section, we present detailed discussion for each experiment that we fully carried out and analyzed (presented in the main core of this article). The focus of this section is to highlight potential issues and alternative explanations that could have led to negative results.

**S.2.1. Experiment 1, windows experiment**

Initially designed as a training step for another experiment (see the previous section for details), cleaners were "overtrained" to locate four food items on a plate. Over time, it became evident that this could serve as a useful first step in examining whether cleaners could efficiently find food items when they were not always directly visible. In the extreme, solving the task as efficiently as in the absence of windows may require some level of object permanence (knowing that the items out of sight still exist) and working memory in order to avoid revisiting emptied locations. Alternatively, an efficient performance could be achieved by developing a routine, visiting the four windows always in the same order. We did not have to investigate this simpler mechanism as the performance of cleaners dropped significantly with respect to all three measures, i.e. time until completion, mistakes and extra looks. A weakness of the experiment is that one cannot make a prediction how many mistakes and looks should occur if cleaners have zero WM, and hence one cannot exclude that the observed performance is best explained with cleaners having a low level of WM, or the decline in performance being entirely linked to poor object permanence.

As for the small differences in performance between the plates, caution is needed when interpreting these results. The patterns and colors on the fish plate could have acted as cues, in contrast to a uniformly white plate, without necessarily being linked to ecological relevance. If one were interested in studying the issue in detail, fish of various colors could be used, and a computer program could help design plates with the overall same color distribution and eye and fins in unlikely places as control stimuli.

**S.2.2. Experiment 2, Working Memory box: the movable arena**

While the failure of cleaners in our paradigm could be due to a lack of WM, we acknowledge that the experimental design is rather complicated. Potentially, swimming back through the corridors may have been a serious distraction, and distractions reduce WM efficiency. We had several days of training to familiarize cleaners with the procedure but a future experiment might benefit from more days of training and testing.

Another potential problem for the cleaners could have been that the plate was uniformly white. Marking the four entrances with four different colors and patterns may have helped to remember ‘who’ was visited rather than ‘where’ was visited. Based on the cleaners’ ecology, we would generally expect that presenting a task as ‘social’ rather than spatial would improve performance.

Finally, if cleaners were to have rather limited WM capacities, the experimental design would have low power to detect such capacities. This is because the design is such that subjects could show a WM capacity of one, two or even three items, depending on whether they perform above chance in their second, third or fourth choice. If cleaners had a WM capacity of 1, they should perform above chance only in their second choice. However, the probability of choosing correctly by chance for the second choice is already 75%. While it is difficult under these conditions to perform significantly above chance, we think that we had a decent sample size, and the mean performance was almost exactly at chance levels, with no individual appearing to perform clearly better. But there is also the possibility that such a high chance level may have prevented cleaners from making the effort to memorize their initial choice.

Despite these methodological challenges, we think that the movable arena remains a promising paradigm for investigating WM. One of its key advantages is that, once subjects are adequately trained, the time interval between choices can be easily manipulated by adjusting the length of the testing area. Additionally, the setup requires minimal intervention from the experimenter, in contrast to paradigms that involve the physical movement of plates or objects within the water, which is critical for minimizing disturbance during WM tests. Also, the paradigm allows for modifications such as adding colors or other types of information to the compartments, thus enabling the modification of various test conditions. This methodological flexibility offers intriguing possibilities for investigating different cognitive dimensions of WM in animals.

**S.2.3. Experiment 3, two plates experiment**

An important aspect of the experimental design was that half of the cleaners would obtain a second food item if choosing the same plate type, while the other half would obtain a second food item if choosing the different plate type. Thus, the design was very much a (delayed) match-to-sample or odd-one-out experiment. As we had expected, based on the logic that a recently cleaned client first needs to get reinfected to offer again food, cleaners exhibited a spontaneous preference for the different plate type during the first trials, i.e. before they could learn their specific tasks, which could reflect a familiarity process (although we did not specifically assess this possibility). It is, therefore, an interesting question why this preference disappeared in the group of cleaners for which it was the correct choice.

The only potential explanation we can offer concerns a detail in the reward scheme. Research on guppies shows that offering always access to both the rewarding and the non-rewarding options speeds up associative learning in a simultaneous two-choice task (comparing Triki et al., 2022 and Triki et al., 2024). We have recently used the method on cleaners for associative learning with similar success (unpublished data). We, therefore, decided for the current study that the cleaners would also be allowed to access both plates in each trial, irrespective of whether their first choice was right or wrong. However, what might have happened in this more complex task – where they needed to update information about the plates after each trial – the same reward scheme might have caused cleaners to abandon the higher cognitive efforts to make the correct choice, and instead simply inspect both plates when necessary. Precedence for such an interpretation comes from work on common marmosets (Burkart & Heschl, 2006; Schubiger et al., 2016). In the first study, an experimenter gave visual cues (like gaze or pointing) to indicate a location where a food item was hidden. If only two hiding locations existed, the monkeys performed at chance levels and hence obtained food in about 50% of trials. However, when there were nine hiding locations, monkeys performed significantly above chance (Burkart & Heschl, 2006). In the second study, an object permanence task testing the performance of marmosets and squirrel monkeys with different delays, subjects again performed better if confronted with nine locations rather than with two locations (Schubiger et al., 2016). The authors concluded that the monkeys only paid close attention when it was perceived as worth-while the effort. In our study, the costs of being inattentive was minimal as the cleaners would always obtain the food, and hence just lose few seconds if inspecting the wrong plate first.

**S.2.4. Experiment 4, dynamic two plates experiment**

We consider this experiment to capture a key element of the cleaners’ ecology, namely that they interact with different clients in rapid successions, and cleaning one client means that it is depleted for some time. Therefore, choosing the other client is what cleaners also do in nature. The main difference between nature and the experiment is that in nature, a cleaner would swim directly from one client to the other, rather than having to wait until the two clients are at equal distances. The fact that the two plates are always visible is a strong advantage against a potential bias due to the temporary disappearance of a plate, where cleaners could have associated the similar plate with a brand-new one.

As to why cleaners might have WM but still failed in the experiment, we can only offer partly the same potential explanation as we gave for experiment 3. The complexity of the task, requiring prolonged attention in order to perform above chance, may have triggered cleaners to be inattentive as they would receive a food reward in 50% of cases anyway (though not 100% as we removed the plate that was not chosen, contrary to experiment 3).

For colleagues interested in using the design for WM tests on their study species, we want to briefly develop on why we used four different plates. This was because if cleaners had performed above chance level in the pairwise design, we could have increased complexity by presenting three, or even four plates simultaneously. In that case we would have placed three plates out of reach, then move two plates forward so that the cleaner can eat the items off them, move them back into place, and then lift the barrier to give cleaners access to all three plates. The same logic can be applied to four plates. By varying the number of plates, one can vary the number of items that cleaners must keep in mind in order to be able to solve the task. Thus, the task provides a design that allows to test WM capacities in a quantitative way, rather than merely for presence/absence.
